# Peripheral oxytocin treatment ameliorates obesity by reducing food intake and visceral fat mass

**DOI:** 10.18632/aging.100408

**Published:** 2011-12-17

**Authors:** Yuko Maejima, Yusaku Iwasaki, Yui Yamahara, Misato Kodaira, Udval Sedbazar, Toshihiko Yada

**Affiliations:** ^1^ Department of Physiology, Division of Integrative Physiology, Jichi Medical University School of Medicine, 3311-1 Shimotsuke, Tochigi 329-0498, Japan; ^2^ Department of Developmental Physiology, Division of Adaptation Development, National Institute for Physiological Sciences, Okazaki, Aichi 444-8585, Japan

**Keywords:** oxytocin, visceral obesity, fatty liver, metabolic syndrome, impaired glucose tolerance, feeding, energy expenditure

## Abstract

Recent studies suggest that oxytocin (Oxt) is implicated in energy metabolism. We aimed to explore acute and sub-chronic effects of peripheral Oxt treatment via different routes on food intake and energy balance. Intraperitoneal (ip) injection of Oxt concentration-dependently decreased food intake in mice. Ip Oxt injection induced c-Fos expression in the hypothalamus and brain stem including arcuate nucleus (ARC), paraventricular nucleus (PVN) and nucleus tractus solitarius (NTS). Subcutaneous (sc) injection of Oxt suppressed food intake in normal and high fat diet-induced obese (DIO) mice. Daily sc injection of Oxt for 17 days in DIO mice reduced food intake for 6 days and body weight for the entire treatment period and additional 9 days after terminating Oxt. Oxt infusion by sc implanted osmotic minipumps for 13 days in DIO mice reduced food intake, body weight, and visceral fat mass and adipocyte size. Oxt infusion also decreased respiratory quotient specifically in light phase, ameliorated fatty liver and glucose intolerance, without affecting normal blood pressure in DIO mice. These results demonstrate that peripheral Oxt treatment reduces food intake and visceral fat mass, and ameliorates obesity, fatty liver and glucose intolerance. Peripheral Oxt treatment provides a new therapeutic avenue for treating obesity and hyperphagia.

## INTRODUCTION

Oxytocin (Oxt) is a well known neurohypophysial hormone, playing an essential role in mammalian labor and lactation [1], via its peripheral action. Recent reports have documented the effects of Oxt in the central nervous systems, including maternal nurturing, social attachment [2, 3], and promotion of learning and memory [4]. Moreover, the physiological role of Oxt in the energy metabolism has recently been reported. Fasting decreases and refeeding increases Oxt mRNA in PVN [5]. Intracerebroventricularly (ICV) injection of Oxt reduces food intake [6], while ICV injection of Oxt antagonist increases food intake [6, 7]. The central Oxt-induced anorexia requires the melanocortin pathway [8]. Furthermore, therapeutic potential was suggested by the finding that ICV injection of Oxt suppresses food intake in Zucker fatty rats, a leptin-resistant obese model [8], indicative of the ability of Oxt to evoke its anorectic neural pathway independently of leptin.

A report by Arletti et al. (1989) [6] first documented anorexia induced by peripheral Oxt. The very recent publications have also reported that intraperitoneal (ip) injection of Oxt suppresses food intake [7, 9]. In an apparent discrepancy, mice deficient of Oxt or Oxt receptor developed late-onset obesity without changing food intake [10, 11]. These reports taken together suggest that Oxt is a catabolic, as well as anorectic, peptide, and that Oxt could exert one or both of these effects depending upon the route and/or period of Oxt treatment or deficiency. Mechanisms underlying anorectic and catabolic effects of peripheral Oxt are largely unknown. In this study, we aimed to clarify the acute vs. subchronic effects of peripheral Oxt on food intake and energy balance and the dependency of these effects on the route and method of treatment, and to identify the brain regions stimulated by peripheral Oxt.

We found that ip and sc Oxt treatment acutely suppresses food intake, and that subchronic Oxt infusion by sc implanted osmotic minipumps reduces BW, visceral fat mass and adipocyte size as well as ameliorating fatty liver and glucose intolerance in high fat diet (HFD)-induced obese mice, indicating an anti-obesity effect of Oxt infusion. We also found that many nuclei of the hypothalamus and brain stem express c-Fos in response to peripheral Oxt.

## RESULTS

### Ip injection of Oxt suppresses food intake and induces c-Fos expression in the hypothalamus and brain stem

**Figure 1:**
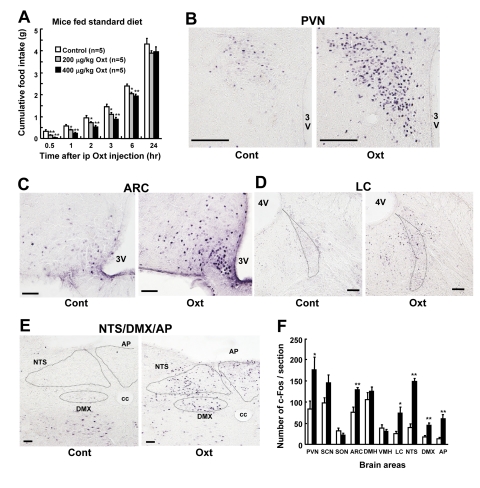
Ip injection of Oxt suppresses food intake and activates neurons in the hypothalamus and brain stem in standard diet fed mice (**A**) Food intake after ip injection of Oxt. White, gray and black bars indicate saline (n = 5), 200 μg/kg dose of Oxt (n = 5) and 400 μg/kg dose of Oxt (n = 5), respectively. (**B-E**) C-Fos expression in the PVN (**B**), ARC (**C**), LC (**D**), and the region including NTS, DMX and AP (**E**) after saline (each left panel) or 400 μg/kg dose of Oxt (each right panel) injection. cc: central canal. (**F**) Number of c-Fos immunoreactive neurons in the feeding-related areas of the hypothalamus and brain stem after injection of 400 μg/kg dose of Oxt (filled bars) or saline (open bars). n = 5 for Oxt and for control. *p < 0.05, **p < 0.01.

Ip injection of Oxt at 200 μg/kg and 400 μg/kg significantly suppressed cumulative food intake for 0.5 to 6 hr after injection, compared with vehicle injection (Figure 1A). Furthermore, ip injection of Oxt at 400 μg/kg induced c-Fos expression in the paraventricular nucleus (PVN) (Figures 1B) and arcuate nucleus (ARC) of the hypothalamus (Figures 1C), and in locus coeruleus (LC) (Figures 1D), nucleus tractus solitarius (NTS), dorsal motor nucleus of vargus nerve (DMX) and area postrema (AP) of the brain stem (Figures 1E). In contrast, Oxt did not significantly induce c-Fos expression in the suprachiasmatic nucleus (SCN), supraoptic nucleus (SON), dorsomedial hypothalamic nucleus (DMH) and ventromedial hypothalamic nucleus (VMH) of the hypothalamus. Numbers of c-Fos in these areas are shown in Figure 1F.

### Peripheral Oxt injection daily decreases food intake and body weight

**Figure 2:**
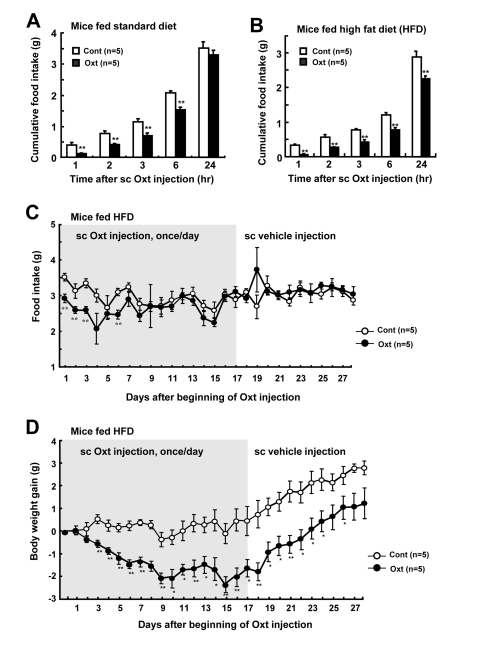
Daily sc injection of Oxt decreases food intake and body weight (**A, B**) Cumulative food intake after 1,600 μg/kg dose of sc injection of Oxt in standard chow fed mice (**A**) and HFD-induced obese mice (**B**). n = 5 for Oxt and for control. (**C, D**) Daily food intake (**C**) and body weight gain (**D**) under sc Oxt injection (1,600 μg/kg), once a day. Gray areas in Figures C and D indicate the phase of Oxt injection (1-17 days). Sc Oxt injection was terminated and switched to sc vehicle injection at day 18. n = 5 for Oxt and for control. *p < 0.05, **p < 0.01.

Single subcutaneous (sc) injection of Oxt at 1,600 μg/kg suppressed food intake in mice fed standard diet (Figure 2A) and high fat diet (HFD) (Figure 2B). Repetitive sc injection of Oxt (1,600 μg/kg) once a day for 17 days in HFD-induced obese mice decreased daily food intake for up to day 6 (Figure 2C). At day 7 and later, there was no significant difference between control and Oxt groups in food intake. BW gain also decreased sharply until day 9 after beginning of Oxt injection, followed by a stable period without rebound increase (Figure 2D). At day 17 when Oxt injection was terminated, BW gain was −1.6 ± 0.4 g, which corresponded to −3.6% of the BW of day 0. BW gain didn't recover to the level of control during the period of Oxt injection. Following the switch from Oxt sc injection to vehicle sc injection at day 17, the reduced BW gain in Oxt group continued to be significantly observed for 9 days until day 26 in the absence of Oxt, suggesting a legacy effect (Figure 2D).

### Chronic Oxt infusion decreases BW, food intake and abdominal fat mass

**Figure 3:**
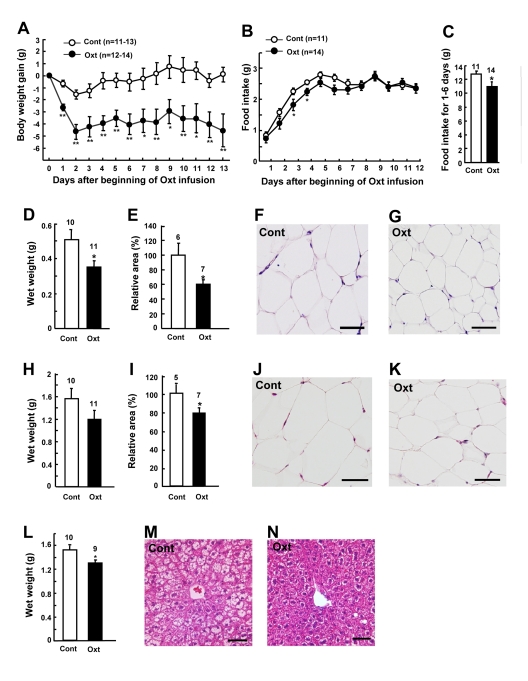
Chronic Oxt infusion decreases body weight, food intake and visceral fat mass (**A, B**) Body weight gain (**A**) and daily food intake (**B**) after implantation of Oxt (1,600 μg/kg/day) infusion minipump. (**C**) Cumulative food intake for day 1 through day 6 is calculated from Fig. 3B. (**D-G**) Wet weights (**D**) and relative areas (**E**) of mesenteric fat in control and Oxt infused mice. Microphotographs of mesenteric fat in control (**F**) and Oxt infused (**G**) mice. (**H-K**) Wet weights (**H**) and relative areas (**I**) of epididymal fat in control and Oxt treated mice. Microphotographs of epididymal fat in control (**J**) and Oxt treated (**K**) mice. Relative areas of adipocytes were calculated from histological section in mesenteric and epididyamal fat. (**L-M**) Wet weights of liver in control and Oxt treated (**L**) mice. Microphotographs of control (**M**) and Oxt infused (**N**) mice. Scale bars in all photomicrographs indicate 50 μm. *p < 0.05, **p < 0.01.

Chronic Oxt infusion (1,600 μg/kg/day) by osmotic minipumps significantly decreased BW gain (Figure 3A) in HFD-induced obese mice. BW decreased rapidly for the first 2 days, followed by a stable reduction (Figure 3A). BW gain was -4.6 ± 1.2 g at the end of Oxt infusion, which corresponded to −13% of the BW of day 0. Food intake decreased for the first 6 days, with statistically significant difference obtained at days 3 and 4 (Figures 3B and C).

On day 14 after sc implantation of minipumps, wet weights and cross section areas of mesenteric fat (Figures 3D and E) and epididymal fat (Figures 3H and I) were decreased in Oxt-treated group, compared with vehicle-treated group. Adipocyte sizes in mesenteric fat (Figures 3F and G) and epididymal fat (Figures 3J and K) were smaller in Oxt-treated group than in vehicle-treated group. In HFD-induced obese mice, liver weight was increased and fat was accumulated in hepatocytes, and Oxt treatment reduced the liver weight and corrected the fat accumulation in hepatocytes (Figures 3L, M and N).

### Chronic Oxt infusion promotes fat usage and improves glucose tolerance

**Figure 4:**
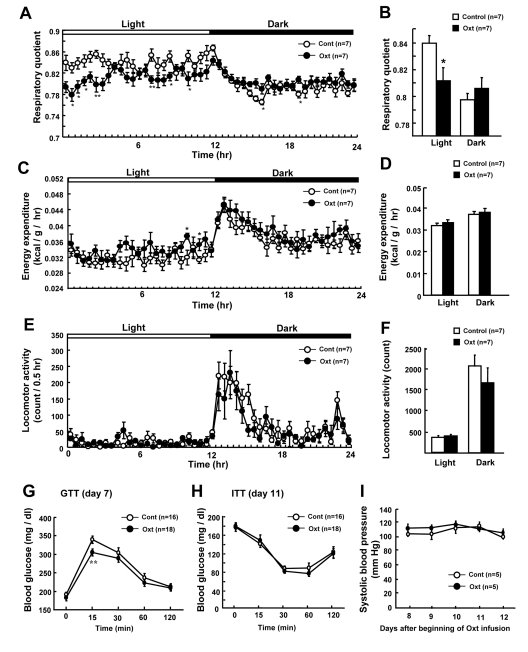
Chronic Oxt infusion promotes use of fat and improves glucose tolerance (**A, B**) Effect of chronic Oxt infusion on the time course of respiratory quotient (RQ) (**A**) and average RQ in the light and dark phases (**B**). Each value in panel A represents the average of 6 measurement points during 0.5 hr. (**C, D**) Effect of chronic Oxt infusion on the time course of energy expenditure (EE) (**C**) and its average values for the light and dark phases (**D**). Each value in C represents the average of 6 points measured in 0.5 hr (**C**). (**E, F**) Effect of chronic Oxt infusion on the time course of cumulative locomotor activity every 0.5 hr (**E**) and its average values in the light and dark phases (**F**). (**G, H**) Blood glucose levels during ip GTT (2 g/kg glucose injection, n = 18 for Oxt and 16 for control) at day 7 (**G**), and those during ITT (1 IU/kg human insulin injection, n = 18 for Oxt and 16 for control) at day 11 (H). (**I**) Systolic blood pressure for 5 days from day 8 to 12 after beginning of Oxt infusion. n = 5 in each group. *p < 0.05, **p < 0.01.

In order to identify the effect of Oxt on different components of energy balance, respiratory quotient (RQ) (Figures 4A and B), energy expenditure (EE) (Figures 4C and D) and locomotor activity (Figures 4E and F) were measured. Oxt infusion decreased RQ specifically in the light phase (Figures 4A and B). EE was slightly increased at several time points in Oxt infusion group (Figure 4C) compared to control group, although no significant difference was observed between two groups when averaged for light and dark phases (Figure 4D). Locomotor activity did not differ between control and Oxt infusion groups (Figures 4E and F).

To assess the effect on glucose metabolism, GTT (Figure 4G) and ITT (Figure 4H) were performed in 4 hr fasted mice. Fasting blood glucose level was slightly elevated by HFD similarly in control and Oxt infusion groups. In GTT, the blood glucose level at 15 min after ip glucose injection was lower in mice receiving Oxt infusion compared with those receiving vehicle infusion (Figure 4G). On the other hand, glucose levels were similar between control and Oxt infusion groups in ITT (Figure 4H).

In order to check the effect of chronic Oxt infusion on the blood pressure, systolic blood pressure was measured from day 8 to 12 (Figure 4I). The systolic blood pressure (Figure 4I) and heart rate (data not shown) were normal in HFD-induced obese mice and they were not different between Oxt and vehicle infusion groups in the period from day 8 through day12.

## DISCUSSION

We found that ip injection of Oxt suppresses food intake in a concentration-dependent manner, confirming previous report [6], and induces c-Fos expression in PVN, ARC, LC and dorsal vagal complex (NTS, DMX, and AP). We can speculate two pathways through which peripherally injected Oxt induces anorexia; the blood-brain barrier (BBB) to ARC pathway and the vagal afferent pathway. ARC is considered the first order center that senses peripheral signals including hormones that penetrate through BBB. It was reported that 0.002% of peripherally injected Oxt reaches central nervous system [12], and that ARC expresses Oxt receptor mRNA [13]. Our result of remarkable expression of c-Fos in ARC after ip injection of Oxt suggests that ip Oxt injection causes anorexia partly by activating anorectic neurons in ARC, including POMC neurons. In this study, ip Oxt injection also induced marked expression of c-Fos in NTS, where the vagal afferent nerve terminates. Therefore, the vagal afferent nerve appears to be the possible alternative pathway for peripheral Oxt, as previously suggested [14]. It is established that peripheral injection of cholecyctokinin (CCK-8) induces anorexia at least partly via vagal afferent nerve [15]. CCK-8 injection induces c-Fos expression in NTS, AP, LC and PVN [16], the brain regions that were activated by peripheral Oxt injection in the present study. It is possible that peripheral injection of Oxt induces anorexia via BBB-ARC and/or vagal afferent nerve routs. However, further studies are definitely needed to identify the neuronal pathway for anorexia induced by peripheral injection of Oxt.

Chronic Oxt infusion by osmotic minipump decreased food intake, BW, and abdominal fat mass, in consistent with the very recent report on rats [17]. Oxt treatment decreased RQ specifically in the light phase (Figures 4A and B), without significantly altering locomotor activity (Figures 4E and F). Food intake in the light phase was not different between control and Oxt infusion groups (data not shown), suggesting that the reduction of RQ does not result from change in food intake. These results indicate that Oxt promotes use of fat as energy substrate in the light phase. Oxt receptors are expressed in adipocytes [18]. It has recently been reported that Oxt induces lipolysis and β-oxidation of fatty acids, and that Oxt reduces fat mass via its direct effect on adipocytes [17]. Taken together, it appears that peripheral Oxt reduces fat mass by multiple mechanisms, including central anorectic action, centrally-mediated activation of sympathetic nerve innervating fat tissue, and its direct peripheral effect on adipocytes, although the action mechanism of peripheral Oxt remain to be further studied.

This is the first report that Oxt improves fatty liver. Fatty liver impairs glucose and lipid metabolism, thereby promoting type 2 diabetes, metabolic syndrome and cardiovascular disease, and also increases the risk of cirrhosis and hepatic cancer [19]. Inhibition of accumulation of fat in liver contributes to prevention of these diseases. Regarding the mechanisms through which peripherally administered Oxt acts on the liver, it has been reported that Oxt directly affects the glycogen synthesis in hepatocytes [20] and that Oxt evokes central regulation of hepatic cholesterol metabolism [21], suggesting possible involvement of direct effect of Oxt and/or indirect action of Oxt mediated by the central nervous system. The fatty liver-correcting effect provides peripherally administrated Oxt with an advantage as a potential agent to treat obesity and metabolic syndrome, although the underlying mechanisms remain to be clarified.

Oxt improved glucose tolerance in GTT, without significantly altering insulin sensitivity in ITT and fasting glucose levels. These results suggest that Oxt could stimulate insulin secretion in response to glucose. In fact, it was reported that Oxt promotes insulin secretion via vagal cholinergic neurons innervating β-cells [22] and that Oxt directly stimulates insulin release from mice islets via phosphoinositide turnover and activation of protein kinase C [23].

We found that chronic sc Oxt infusion by osmotic minipump for 13 days reduced BW of HFD-induced obese mice by -13%, which is greater than the expected level for anti-obesity agents (−10%). Furthermore, the BW-reducing effect by daily sc injection of Oxt lasted for 9 days after termination of Oxt injection, suggesting a legacy effect. Sc injection is an useful route for drug delivery. In the present study, infusion of Oxt at 1,600 μg/day, which elicited anti-obesity effect, was without effect on the normal level of systolic blood pressure, as an indication of safety. It was previously reported that Oxt increased sympathetic tone [10, 24], an effect which could be linked to an increase in blood pressure [24]. However, Oxt at the concentration used in our experiment had no significant effect on the levels of urinary catecholamines (data not shown), suggesting the lack of effect on sympathetic tone.

This study demonstrated that chronic peripheral Oxt infusion ameliorates obesity in the HFD-induced obese mice by reducing food intake and visceral fat mass and possibly by promoting fat usage as the energy substrate, and that these changes are associated with correction of fatty liver and amelioration of glucose intolerance, without altering the normal blood pressure level of this model. Thus, hyperphagia, visceral obesity, fatty liver and hyperglycemia were all ameliorated by Oxt. This study has revealed a profound and stable anti-obesity action of chronic peripheral Oxt infusion, providing the basis for its application to treat human subjects with obesity, hyperphagia and type 2 diabetes.

## METHODS

### Animals

Male C57BL/6J mice aged 6 weeks were obtained from Japan SLC (Japan). Animals were maintained on a 12-hr light/dark cycle and allowed *ad libtum* access to water and to either a standard diet (CE-2; Clea, Osaka, Japan) or a high fat diet (HFD) containing 32% kcal from fat (HFD32; Clea, Osaka, Japan) for 8 weeks. Experimental procedures and care of animals were carried out according to the Jichi Medical University Institute of Animal Care and Use Committee.

### Measurements of food intake under single oxytocin injection

Following deprivation of food for 2 hr before the dark phase, animals were intraperitoneally (ip) injected with 200 or 400 μg/10ml/kg Oxy (Peptide Institute, Osaka, Japan) or subcutaneously (sc) injected with 1,600 μg/5ml/kg Oxy at the beginning of dark phase. Then cumulative food intake for the following 0.5, 1, 2, 3, 6 and 24 hr was measured. Control mice were injected with vehicle, sterile saline (0.9% NaCl).

### Measurements of food intake and body weight under daily Oxt injection

HFD-induced obese mice (43.9 ± 0.8 g BW) were sc injected once a day with 1,600 μg/5 ml/kg Oxt or vehicle for 17 days, followed by vehicle injection for 10 days in both test and control groups. Injection was executed at 2 hr before the dark phase, when BW and food intake were measured.

### Measurements of food intake, body weight, and fat weight under chronic Oxt infusion

HFD-induced obese mice (35.5 ± 0.8 g BW) received sc infusion of Oxt at 1,600 μg/kg/day or vehicle over 14 days using osmotic minipumps (Alzet, model 2002, CA), as previously described [25]. At day 14 after beginning of oxytocin infusion, weights of isolated liver, mesenteric fat and epididymal fat were measured. Small pieces of these tissues were fixed in 4% paraformaldehyde and embedded in paraffin. Five μm sections were stained with hematoxylin and eosin. Size (relative area) of adipocyte was measured by NIH image software (Image J 1.44p, National Institute of Health).

### Measurements of c-Fos

Ninety minutes after ip injection of Oxt (400 μg/kg), mice were transcardially perfused, and coronal sections at 120 μm interval were processed for c-Fos immunoreactivity as described previously [26]. Anti-c-Fos antisera sc-52 (Santa Cruz, CA, 1:5,000) were used as the primary antibody.

### Measurements of respiratory quotient, energy expenditure, and locomotor activity

Oxygen consumption (VO_2_) and carbon dioxide production (VCO_2_) were measured by using indirect calorimetry system (Oxymax; Colombus Instrument, OH), as previously described [25]. Briefly, HFD-induce obese mice, at day 9-14 after beginning of Oxt infusion, were placed in individual small acryl calorimeter chambers for mouse, with free access to HFD and water. After 20-24 hr of adaptation, VO_2_ and VCO_2_ were measured in individual mouse for 1 min at 5 min intervals over a 24 hr period with an air flow at 0.6 l/min. The respiratory quotient (RQ) was calculated as the volume of VCO_2_ per volume of VO_2_, in ml/min. Energy expenditure (EE) was calculated as EE = (3.85 + 1.232 × RQ) × VO_2_. Locomotor activity was estimated by the number of infrared beams broken in both X and Y directions using an activity monitoring system (ACTIMO-100; Shinfactory, Fukuoka, Japan) combined with individual calorimeter chambers.

### Tests of glucose metabolism

For glucose tolerance test (GTT), 4-hr-fasted HFD-induce obese mice were ip injected with glucose (2 g/kg). For insulin tolerance tests (ITT), 4-hr-fasted HFD-induced obese mice were ip injected with human insulin (1 IU/kg). Glucose levels in blood samples obtained from tail vein were determined by using Glucocard (Arkray, Kyoto, Japan). GTT was performed at day 7 and ITT at day 11 after beginning of Oxt infusion.

### Measurements of systolic blood pressure

Systolic blood pressure was determined by photoelectric/oscillometric tail-cuff method (Model MK-2000; Muromachi Kikai Co., LTD, Tokyo, Japan). Mice were habituated with measuring systolic blood pressure for 5 days prior to Oxt infusion. The data of systolic blood pressure were obtained from day 8, when rats were recovered from surgery of minipump implantation. The average value of 5 measurements was used as a datum. Systolic blood pressure was measured at 13:00 to 15:00 (light phase) during 5 days.

### Statistical analysis

All data were presented mean ± SEM. The statistical analysis of experimental data for two groups was carried out with unpaired t-test. One-way ANOVA followed by Bonferroni's Multiple Range tests were used to compare multiple test groups.

## References

[R1] Kiss A, Mikkelsen JD (2005). Oxytocin-anatomy and functional assignments: a minireview. Endocr. Regul..

[R2] Donaldson ZR, Young LJ (2008). Oxytocin, vasopressin, and the neurogenetics of sociality. Science.

[R3] Neumann ID (2008). Brain oxytocin: A key regulator of emotional and social behaviors in both females and males. J. Neuroendocrinol..

[R4] Tomizawa K, Iga N, Lu YF, Moriwaki A, Matsushita M, Li ST, Miyamoto O, Itano T, Matsui H (2003). Oxytocin improves long-lasting spatial memory during motherhood through MAP kinase cascade. Nature Neurosci.

[R5] Tung YC, Ma M, Piper S, Coll A, O'Rahilly S, Yeo GSH (2008). Novel leptin-regulated genes revealed by transcriptional profiling of the hypothalamic paraventricular nucleus. J. Neurosci..

[R6] Arletti R., Benelli A., Bertolini A. (1989). Influence of oxytocin on feeding behavior in the rat. Peptides.

[R7] Zhang G, Cai D (2011). Circadian intervention of obesity development via resting stage feeding manipulation or oxytocin treatment. Am J Physiol Endocrinol Metab.

[R8] Maejima Y, Sedbazar U, Suyama S, Kohno D, Onaka T, Takano E, Yoshida N, Koike M, Uchiyama Y, Fujiwara K, Yashiro T, Horvath TL, Dietrich MO, Tanaka S, Dezaki K, Oh-I S, Hashimoto K, Shimizu H, Nakata M, Mori M, Yada T (2009). Nesfatin-1-regulated oxytocinergic signaling in the paraventricular nucleus causes anorexia through a leptin-independent melanocortin pathway. Cell Metab.

[R9] Morton GJ, Thacher BS, Reidelberger RD, Ogimoto K, Wolden-Hanson T, Baskin DG, Schwartz MW, Blevins J (2011). Peripheral oxytocin suppresses food intake and causes weight loss in diet-induced obese rats. Am J Phyiol Endocrinol Metab.

[R10] Camerino C (2009). Low sympathetic tone and obese phenotype in oxytocin-deficient mice. Obesity.

[R11] Takayanagi Y, Kasahara Y, Onaka T, Takahashi N, Kawada T, Nishimori K (2008). Oxytocin receptor-deficient mice developed late-onset obesity. Neuroreport.

[R12] Mens WB, Witter A, Van Wismersma Graidanus TB (1983). Penetration of neurophyseal hormones from plasma into cerebrospinal fluid (CSF): half-times of disappearance of these neuropeptides from CSF. Brain Res.

[R13] Ostrowski N (1998). Oxytocin receptor mRNA expression in rat brain: implications for behavioral integration and reproductive success. Psychoneuroendocrinol.

[R14] Schwartz GJ (2006). Integrative capacity of the caudal brainstem in the control of food intake. Phil Trans R Soc B.

[R15] South EH, Ritter RC (1988). Capsaicin application to central or peripheral vagal fibers attenuates CCK satiety. Peptides.

[R16] Mönnikes H, Lauer G, Arnold R (1997). Peripheral administration of cholecystokinin activates c-fos expression in the locus coeruleus/subcoeruleus nucleus, dorsal vagal complex and paraventricular nucleus via capsaicin-sensitive vagal afferents and CCK-A receptors in the rat. Brain Res.

[R17] Deblon N, Veyrat-Durebex C, Bourgoin L, Caillon A, Bussier AL, Petrosino S, Piscitelli F, Legros JJ, Geenen V, Foti M, Wahli W, Di Marzo V, Rohner-Jeanrenaud F (2011). Mechanisms of the anti-obesity effects of oxytocin in diet-induced obese rats. Plos One.

[R18] Gimpl G, Fahrenholz F (2001). The oxytocin receptor system: structure, function, and regulation. Physiol Rev.

[R19] Monteiro R, Azevedo I (2010). Chronic inflammation in obesity and the metabolic syndrome. Mediators Inflamm.

[R20] Ariño J, Bosch F, Gómez-Foix AM, Guinovart JJ (1989). Oxytocin inactivates and phosphorylates rat hepatocyte glycogen synthase. Biochem J.

[R21] Vanpatten S, Karkanias GB, Rossetti L, Cohen DE (2004). Intracerebroventricular leptin regulates hepatic cholesterol metabolism. Biochem J.

[R22] Björkstrand E, Eriksson M, Uvnäs-Moberg K (1996). Evidence of a peripheral and a central effect of oxytocin on pancreatic hormone release in rats. Neuroendocrinol.

[R23] Gao ZY, Drews G, Henquin JC (1991). Mechanism of the stimulation of insulin release by oxytocin in normal mouse islets. Biochem J.

[R24] Pyner S (2009). Neurochemistry of the paraventricular nucleus of the hypothalamus: Implication for cardiovascular regulation. J Chem Neuroanat.

[R25] Toriya M, Maekawa F, Maejima Y, Onaka T, Fujiwara K, Nakagawa T, Nakata M, Yada T (2010). Long-term infusion of Brain-Derived Neurotrophic Factor reduces food intake nad body weight via corticotrophin-releasing hormone pathway in the paraventricular nucleus of hypothalamus. J Neuroendocrinol.

[R26] Kohno D, Nakata M, Maejima Y, Shimizu H, Sedbazar U, Yoshida N, Dezaki K, Onaka T, Mori M, Yada T (2008). Nesfatin-1 neurons in paraventricular and supraoptic nuclei of the rat hypothalamus coexpress oxytocin and vasopressin and are activated by refeeding. Endocrinol.

